# Trends, Symptoms, and Outcomes of Resectable Giant Mediastinal Tumors

**DOI:** 10.3389/fonc.2022.820720

**Published:** 2022-02-04

**Authors:** Xiaoshun Shi, Xiguang Liu, Xiaoying Dong, Hua Wu, Kaican Cai

**Affiliations:** Department of Thoracic Surgery, Nanfang Hospital, Southern Medical University, Guangzhou, China

**Keywords:** giant mediastinal tumor, mediastinal malignancy, risk prediction model, surgical therapy, global survey

## Abstract

Describing the changes in surgical procedures and factors affecting the surgical outcome of patients who have undergone complete resection of giant mediastinal tumors (GMTs, diameter ≥ 10 centimeters) could improve preoperative decision-making and prognostic evaluations. We accessed data from three sources, which are case reports on surgical treatment of GMTs from PubMed, Web of Science, and EMBASE until June 1, 2019; patients with resected GMT from the Surveillance, Epidemiology, and End Results (SEER) database; and retrospective review of medical records in our institution from 2000 to 2019. The worldwide distribution, clinicopathological characteristics, symptom profile, prognosis of patients with GMT resection, and nomogram for surgical outcome prediction are reported. A total of 242 rare GMT cases from four continents (Asia, North America, South America, and Europe) were included. The median age of the patients was 40 (IQR: 27, range: 13–83) years, and the male-to-female ratio was 1.57:1. Dyspnea, shortness of breath, cough, and chest pain or discomfort were the major symptoms at presentation. The prognosis of benign and low-grade malignant GMTs was superior to that of high-grade malignant GMTs. Tumor malignancy played the most critical role in predicting postoperative survival, followed by longest tumor diameter and a posterior mediastinum location. The findings of this study suggest that the number of successful GMT surgeries has increased over the last decade and describe clinical features of GMTs. Physicians should prioritize tumor malignancy as a leading factor in predicting outcome rather than tumor size.

## Introduction

Giant mediastinal tumors (GMTs) with a diameter greater than or equal to 10 centimeters rarely occur but often impose challenges in the surgical and anesthesia community (1, 2). The clinical presentation and pathological diagnosis vary from case to case (3, 4). Despite these tumors having variable malignant potential, complete resection is considered the first-line treatment in appropriate cases. Although the clinicopathological characteristics of GMTs should be mainly consistent with smaller mediastinal tumors, there is no systemic description of their worldwide demographic characteristics and clinical symptoms or overviews of their mediastinal origin, etiologies, or trends in surgical approaches.

While a better understanding of GMT survival patterns is essential to improving preoperative decision-making and postoperative follow-up schedules, the surgical outcomes are not clear due to the rarity of these tumors. Interestingly, although a large tumor size is a poor prognostic factor in cancer medicine, patients with GMTs tend to have low-grade tumors, and tumor size does not seem to be the most critical prognostic factor. We hypothesized that this entity would have different survival risk factors from smaller tumors due to its slow growing nature.

Given this context, the purpose of this study was to provide the general characteristics of GMTs and survival risk factors by analyzing existing data over the last two decades. The worldwide distribution, clinicopathological characteristics, symptom profile, and prognosis of patients who underwent GMT resection are reported. Additionally, a risk prediction model of GMTs with up to 10-year survival rates was constructed, which may help thoracic surgeons screen high-risk patients and provide better preoperative management.

## Materials and Methods

### Data Sources and Eligibility

The data in this study are from three sources, including from a manual search of the literature on GMTs in PubMed and the Surveillance, Epidemiology, and End Results (SEER, 1975–2016) database and a retrospective retrieval of all data from patients who underwent GMT resection from January 2000 to December 2019 in our center. For the retrospective data in our institute, the requirement for written informed consent for publication was waived given the use of deidentified data.

In this study, GMTs were defined as those that originated from the mediastinum with a diameter greater than or equal to 10 centimeters. From the SEER database, only surgery codes for total surgical removal of the primary site (5) and radical surgery (6) were included. For the literature search, the exclusion criteria were as follows: (I) studies reporting tumors that did not originate from that mediastinum; and (II) potential duplicate reports (the report with the most data was included); (III) studies with incomplete tumor size data (except for apparent size over 10 cm in CT scan); and (IV) reports of incomplete resection. In the current report, patients younger than ten years old were not included.

### Data Collection

For patient demographics, tumor location and diagnosis were collected from the three databases and merged. Because not all tumor grade, disease-free survival (DFS), and overall survival (OS) information were obtained for every patient in the literature, only those with clear descriptions were included. Perioperative data and symptoms were only available from the literature and Nanfang cohort, and the patients were considered symptom-free if no specific symptoms were reported. The extent of tumor invasion, lymph node involvement, and distant metastasis was not considered in this study due to a lack of information.

### Statistical Analysis and Constructing the Risk Prediction Model

Statistical analysis and survival modeling were performed using R (R version 3.5.1). Normally distributed continuous data are presented as the mean ± standard deviation (range), whereas continuous data with a skewed distribution are presented as the median [interquartile range (IQR), range]. The former was compared with Student’s t-test, and the latter was compared with the Mann–Whitney U-test. Chi-squared analysis or Fisher’s exact test was used to compare categorical variables when appropriate. DFS and OS were defined as previously described (7). Kaplan-Meier survival analyses were conducted. Survival curves were plotted using the Kaplan–Meier method, and differences in survival were assessed with log-rank analysis. The risk prediction model was developed and validated according to the guidelines proposed by Grant et al. (8), and a calibration curve was constructed (9). P-values less than 0.05 were considered statistically significant.

## Results

### Demographic and Clinical Characteristics of GMTs

**Figure 1:**
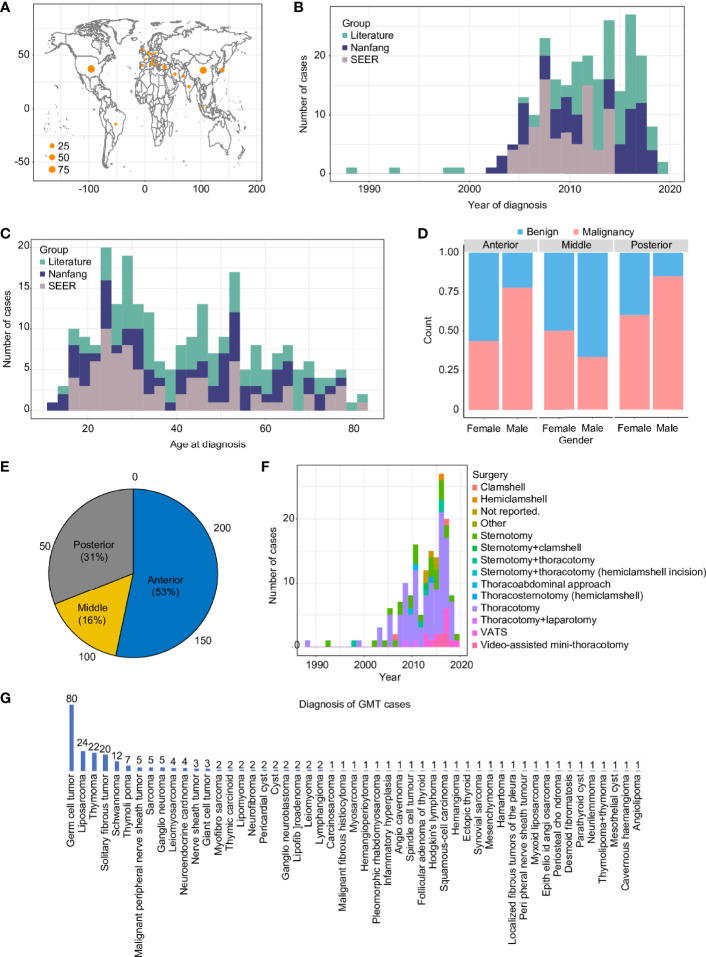
Overview of GMT resections from 1988 to 2019 in three cohorts. GMT distribution in countries around the world **(A)**, in reports from 1988 to 2019 **(B)**, by age **(C)**, by sex **(D)**, by mediastinum location **(E)**, by surgical approach **(F)**, and by pathological diagnosis **(G)**.

A total of 242 GMT cases from three cohorts representing four continents (Asia, North America, South America, and Europe) were included (**Figure S1**). The baseline characteristics were different among the 3 groups (**Table 1**), revealing the clinical diversity of the reported GMT cases. Most of the cases were predominantly from the USA (90) and China (81), followed by Japan (16), Turkey (15), and Italy (11) (**Figure 1A**). After 2020, GMT resection was more frequently documented, possibly due to advancements in surgical techniques (**Figure 1B**). The median age at GMT diagnosis was 40 (IQR: 27, range: 13–83), with two peaks at 25–30 and 45–55 years old (**Figure 1C**). The male-to-female ratio in this study was 1.57:1, with a sex-specific predominance for malignant GMTs (p-value <0.01, Pearson’s chi-squared test with Yates’ continuity correction) (**Figure 1D**). Similar to primary mediastinal tumors in adults (10, 11), the GMTs were also located predominantly in the anterior mediastinum (53%), followed by the posterior (31%), and middle (16%) mediastinum (**Figure 1E**). Although minimally invasive thoracoscopic surgery was performed in some cases, the most common surgical procedures for GMTs were thoracotomy and sternotomy (82%) in the Nanfang and literature cohorts (**Figure 1F**). The average longest diameter in the three cohorts was 15.5 (IQR: 7.5, range: 10–40 cm). The most common etiologies of GMT were germ cell tumors (33%), liposarcoma (10%), and thymoma (10%) (**Figure 1G**).

**Table 1:** Clinical features of the resected GMTs by cohort.

	Literature (N = 92)	Nanfang (N = 69)	SEER (N = 81)	Overall (N = 242)	P value*
**Age at diagnosis**					
Mean (SD)	44.7 (16.6)	42.2 (18.1)	38.8 (18.0)	42.0 (17.6)	
Median [Min, Max]	44.0 [15.0, 82.0]	44.0 [13.0, 78.0]	32.0 [18.0, 83.0]	40.0 [13.0, 83.0]	
**Gender**					
Female	41.0 (44.6%)	38.0 (55.1%)	15.0 (18.5%)	94.0 (38.8%)	
Male	51.0 (55.4%)	31.0 (44.9%)	66.0 (81.5%)	148 (61.2%)	< 0.01
**Location**					
Anterior	47.0 (51.1%)	35.0 (50.7%)	47.0 (58.0%)	129 (53.3%)	
Middle	16.0 (17.4%)	21.0 (30.4%)	28.0 (34.6%)	65.0 (26.9%)	< 0.01
Posterior	29.0 (31.5%)	13.0 (18.8%)	6.00 (7.4%)	48.0 (19.8%)	
**Malignancy**					
Benign	55.0 (59.8%)	29.0 (42.0%)	0 (0%)	84.0 (34.7%)	
Malignant	37.0 (40.2%)	40.0 (58.0%)	81.0 (100%)	158 (65.3%)	< 0.01

*P-values were calculated using Chi-square test for categorical variables to compare the difference between groups.

### Symptomatic Profile of GMTs

**Figure 2:**
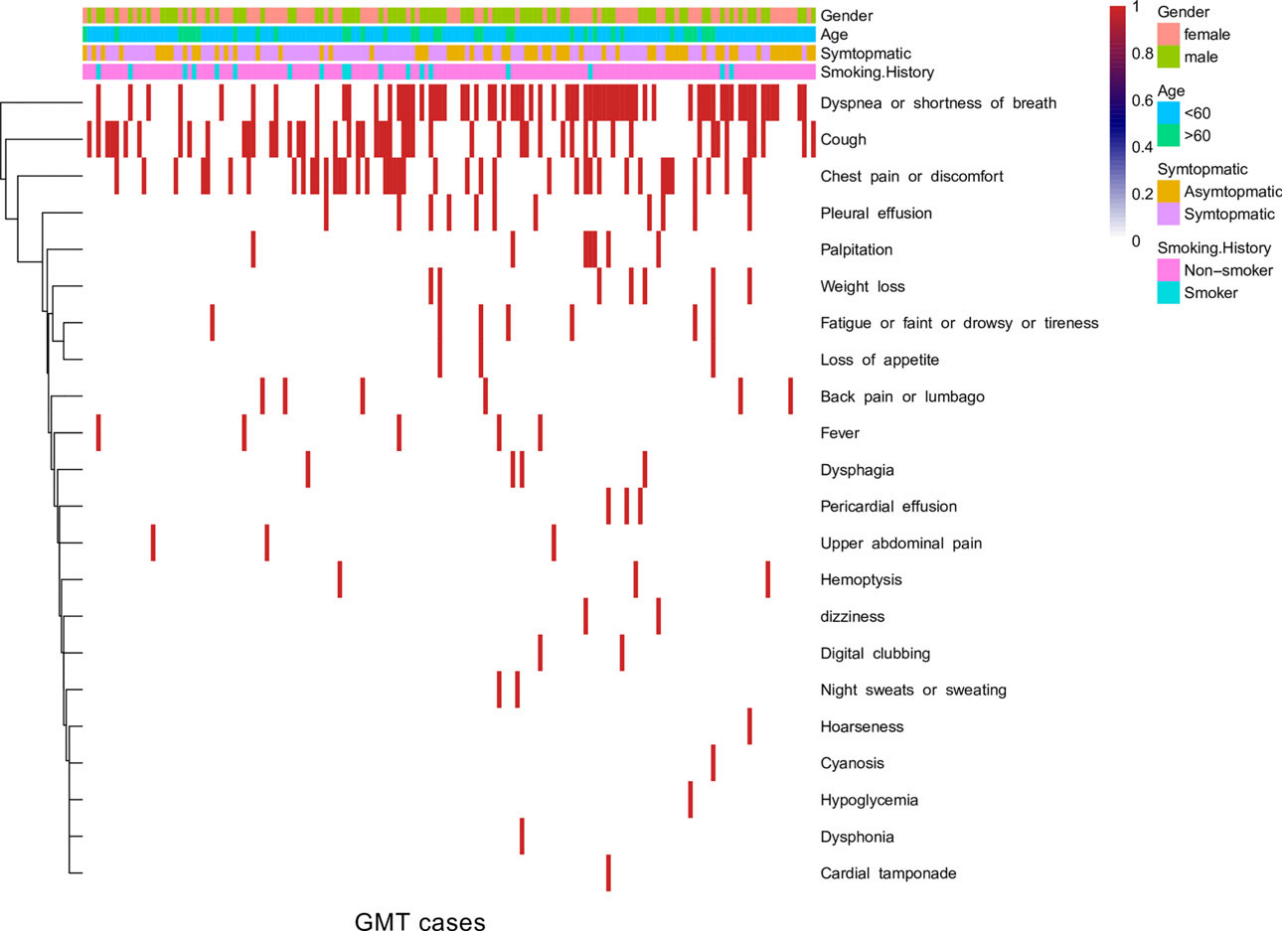
Heatmap of GMT symptoms in the Nanfang and literature cohorts.

The symptoms of GMTs varied from case to case. In the Nanfang and literature cohorts, the ratio of symptomatic-to-asymptomatic presentations was 5:1. Over the study period, dyspnea, shortness of breath, cough, and chest pain or discomfort were the primary symptoms at presentation (**Figure 2**). The presenting symptom was not associated with age at diagnosis (p value = 0.9, Pearson’s chi-squared test) or sex (p value = 0.1, Pearson’s chi-squared test). The presence of symptoms at presentation was not associated with a survival of less than one year (p value = 0.9, chi-squared test), and the number of presenting symptoms was not correlated with survival duration (R^2^ = 0.2).

### Perioperative Outcomes

Approximately 5% of patients underwent piecemeal surgery; otherwise, en bloc resection was performed. The mean operation times in the literature and Nanfang cohort were 230 ± 149 (range: 105–508) and 150 (IQR: 91.25, range: 42–450) min, respectively, and the mean intraoperative blood loss was 823 ± 675 (range: 90–1774) and 200 (IQR: 500, range: 20–4000) ml, respectively. The mean intraoperative transfusion volume was 304 ± 584 (range: 0–3000 ml), with a rate of 66.7%. The Mann-Whitney U-test revealed no significant difference in the length of postoperative stay (days) between the two cohorts, although a skewed distribution was observed (median: 7 vs. 15 days, P = 1). The overall postoperative complication rate was 9%, and the mortality rate within one week was 1%. Of note, the deaths in both cohorts were occurred in cases of acute onset or emergent cases.

### Oncological Outcomes

**Figure 3:**
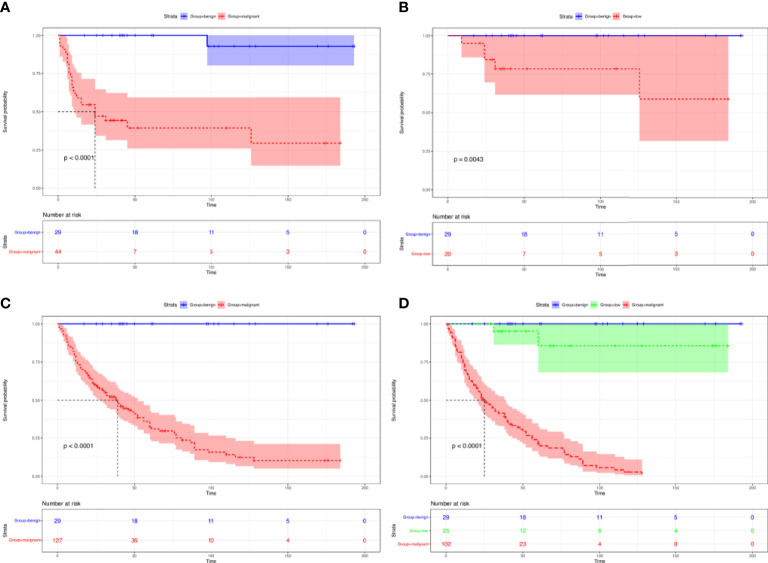
Survival of patients with GMTs who underwent complete resection. Recurrence-free survival in patients with GMTs who underwent complete resection: benign GMTs versus malignant GMTs **(A)**, and benign GMTs versus low-grade malignant GMTs **(B)**. Overall survival in patients with GMTs >10 cm who underwent complete resection: benign GMTs versus malignant GMTs **(C)**, and among benign GMTs and low-grade malignant and malignant GMTs **(D)**.

Consistent with pathological trends, we provided evidence that benign GMTs had an improved recurrence-free survival compared with malignant GMTs (**Figure 3A**) and low-grade GMTs (**Figure 3B**) (P < 0.001). Additionally, better OS was observed for benign GMTs than for malignant GMTs (**Figure 3C**) and low-grade GMTs (**Figure 3D**) (P < 0.001).

**Figure 4:**
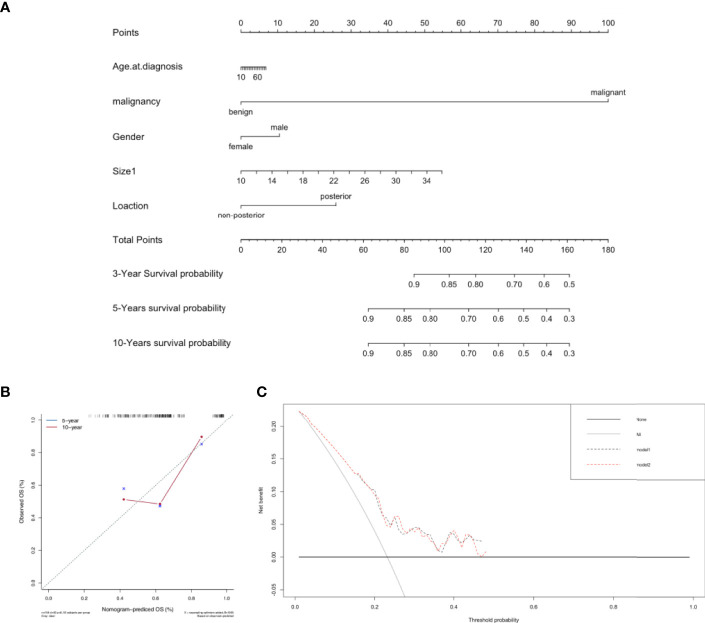
Predictive model for postoperative survival in patients with GMTs who underwent complete resection. **(A)** Postoperative prognostic nomogram for patients with resected GMTs. **(B)** Calibration curves for predicting 5-year and 10-year survival. **(C)** Evaluation of the prediction model by decision-curve analysis.

The prediction model included patient sex, tumor malignancy, longest diameter, and GMT location (**Figure 4A**). The calibration curve suggested that the model had a good predictive performance (**Figure 4B**), and it is widely applicable because it only uses tumor malignancy, longest diameter, location, and sex (**Figure 4C**). We found that tumor malignancy played the most critical role in predicting postoperative survival, followed by tumor longest diameter and posterior mediastinum location.

## Discussion

In recent years, GMTs with a diameter greater than or equal to 10 centimeters have been reported in rare case reports (**Table S1**) (2, 5, 6, 12–91). It is believed that GMTs should follow the basic oncologic principle, and primary surgical resection aided with multimodal therapy is recommended when appropriate. However, direct descriptions of their clinicopathological nature, surgical trends and surgical outcomes and tools for predicting the outcomes of GMTs have yet to be published. To the best of our knowledge, the cases presented in this study are the most extensive report of GMT resection, representing four continents (Asia, North America, South America, and Europe) over the past two decades. Most of the cases were predominantly from the top 3 countries globally by population, namely, the USA (n= 90) and China (n= 81). An increasing trend of successful surgeries was observed, possibly due to the advancement of surgical and anesthetic techniques worldwide.

We first described the features of GMTs in relation to mediastinal tumors, namely, in terms of tumor origin, malignancy, and presenting symptoms. Consistent with a previous report of anterior mediastinal tumors accounting for 50% of all mediastinal masses in children in one report (92) and 44.2% in another (93), 53.3% of the GMTs in this study originated from the anterior mediastinum, confirming the susceptibility of the portion of the mediastinum to tumors. In contrast to the approximately 25% malignancy rate of all mediastinal tumors in both adults and children (92), 65.3% of the GMTs in this study were malignant, accounting for close to 72% of the malignant mediastinal tumors in only children (93). This indirectly reflects that GMTs could have unique malignant characteristics.

In general, malignant lesions are more likely to be symptomatic, and the presenting symptoms were cough, chest pain, fever or chills, and dyspnea (94). Wright and Mathisen (95) reported that more than one-third of mediastinal tumors produce symptoms. In this analysis, 76.4% of the patients with GMTs presented with symptoms, similar to the 70% with symptomatic malignant mediastinal tumors previously reported (10). Based on the first symptomatic heatmap for GMTs, dyspnea, shortness of breath, cough, and chest pain are the major clinical symptoms at presentation and mainly result from tumor compression. Of note, the association of increased GMT risk with the male sex needs further study. Although GMTs are a heterogeneous group of benign and malignant neoplasms, we observed that the predominant diagnosis of GMTs is germ cell tumors.

This study has inherent limitations due to its retrospective design, miscellaneous GMTs all together and use of inconsistent case report data and registry data. First, the Nanfang cohort was from a single-center retrospective study. Not only is the level of evidence not comparable to that from a randomized controlled trial, the potential recall bias during follow-up, surgeon preferences for certain procedures, and updates to instruments could all be confounders. Second, the literature cohort was missing information due to inconsistent report styles, which limited our ability to obtain detailed perioperative and survival data; this warrants further development of a global GMT database. Third, for the SEER cohort, selection bias for malignant disease was obvious. Fourth, the predominant germ cell tumor of GMT in this study based on current data could affect the survival analysis of this entity. We should perform subgroup analysis in the future as more data become available. Fifth, although there is consensus in the society of thoracic surgeons that multidisciplinary treatment and complete surgical resection could improve the prognosis of GMTs, current incomplete data of adjuvant or neoadjuvant treatment, resection status prevent further subgroup analysis for how these factors affect GMTs survival. As such, the development of a consistent report form or a database for GMTs may minimize the amount of missing data and contribute to better understanding of this entity in the mediastinum. Nonetheless, we provided insight based on our collection of GMT cases from across the world in the past two decades.

In conclusion, the surgical treatment and outcome estimations of GMTs is mainly determined by preoperative radiologic images, pathological findings from biopsy, physical status, and the invasion of surrounding anatomical structures. According to this first report of evidence, low-grade GMTs are associated with better survival than high-grade malignant GMTs, suggesting that surgical resection should be recommended for low-grade GMTs. Another proof-of-concept finding is that GMT size is not the most important factor affecting surgical outcome, especially for giant tumors with a slow-glowing nature and unknown pathology. Despite the rareness and surgical challenges of GMTs, the findings of this study provide an oncologic description of GMTs and their associated survival factors. Continuing efforts for consistent reports of such rare cases are needed.

## Data Availability Statement

The raw data supporting the conclusions of this article will be made available by the authors, without undue reservation.

## Ethics Statement

The studies involving human participants were reviewed and approved by Nanfang Hospital. Written informed consent to participate in this study was provided by the patients or participants’ legal guardian/next of kin.

## Author Contributions

KC designed and supervised the study. XS collected and analyzed the data and wrote the manuscript. XD collected the data and revised the manuscript. XL and HW interpreted the results and revised the manuscript. All authors contributed to the article and approved the submitted version.

## Funding

The China Scholarship Council supports the Ph.D. scholarship of XS. The database development work was partially supported by the Research Initiative Fund of Southern Hospital 2018 (C1051325), the Major Science and Technology Planning Project of Guangdong Province (2017B020226005), and the Science and Technology Program of Guangzhou (201903010003).

## Conflict of Interest

The authors declare that the research was conducted in the absence of any commercial or financial relationships that could be construed as a potential conflict of interest.

## Publisher’s Note

All claims expressed in this article are solely those of the authors and do not necessarily represent those of their affiliated organizations, or those of the publisher, the editors and the reviewers. Any product that may be evaluated in this article, or claim that may be made by its manufacturer, is not guaranteed or endorsed by the publisher.
