# Supplementary material for: Trends, Symptoms, and Outcomes of Resectable Giant Mediastinal Tumors
Source: Front Oncol. 2022 Feb 4;12:820720. doi: 10.3389/fonc.2022.820720 (PMC8854276; doi:10.3389/fonc.2022.820720)
Supplement: Supplementary file 1 [file DataSheet_1.docx]

Supplementary Material

Trends, Symptoms, and Outcomes of Resectable Giant Mediastinal Tumors

Xiaoshun Shi, Xiaoying Dong, Xiguang Liu, Hua Wu, Kaican Cai*

Department of Thoracic Surgery, Nanfang Hospital, Southern Medical University, Guangzhou, China

# Supplementary Figures and Tables

## Supplementary Figure

**Supplementary Figure 1.** The study workflow and inclusion criteria.

## Supplementary Table

**Supplementary Table 1.** GMT cases from literature review.
